# Targeting Nfix to fix muscular dystrophies

**DOI:** 10.15698/cst2018.01.121

**Published:** 2017-12-12

**Authors:** Giuliana Rossi, Valentina Taglietti, Graziella Messina

**Affiliations:** 1 Laboratory of Stem Cell Bioengineering, Institute of Bioengineering, Ecole Polytechnique Fédérale de Lausanne (EPFL), Lausanne, Switzerland.; 2 Department of Biosciences, University of Milan, via Celoria 26, 20133, Milan, Italy.

**Keywords:** Nfix, skeletal muscle, satellite cells, muscular dystrophies, slow regeneration, slow oxidative fibers

## Abstract

Muscular dystrophies (MDs) are still incurable heterogeneous diseases, characterized by muscle wasting, replacement by fibrotic tissue, and increasing weakness, which in severe cases, such as Duchenne MD, lead to premature death. MDs are due to mutations encompassing different dystrophin-glycoprotein complex (DGC) genes, which code for structural proteins that anchor the cytoskeleton to the extracellular matrix, thus conferring myofiber stability. All mutations destabilizing this complex result in different MD forms, with varying levels of severity. Independently of the genetic defect, MDs share common hallmarks, characterized by continuous cycles of muscle degeneration, due to lack of structural support during contraction, followed by regeneration cycles by satellite cells (SCs), the canonical myogenic stem cells of adult muscle. However, dystrophic SCs generate new fibres which are also prone to degeneration so that, after many cycles of degeneration/regeneration, this cell population is exhausted and muscle is replaced by connective and adipose tissue. At this stage, any therapeutic intervention is likely to fail.

A shared knowledge in the field is that to be really successful, any therapeutic strategy has to rely on good muscle quality. In fact, there is no available approach able to rescue muscle damage when the muscle tissue has been completely lost and substituted by fibrotic deposits, thus restricting the cohort of patients eligible for clinical trials to the youngest and less compromised individuals. Therefore, among the different approaches, many efforts have been directed to induce hypertrophy and increase muscle regeneration in dystrophic muscle to counteract progressive degeneration. This is obtained at the expense of proliferation and differentiation of SCs, whose pool is large in mice but limited in humans. In fact, these strategies worked in murine muscular dystrophy but the few clinical trials (i.e. myostatin neutralizing antibodies) failed, likely due to a more rapid depletion of the available SC reservoir.

Our recent study (Nat Communications, 8(1):1055) provided a new proof of principle for an innovative therapeutic approach to treat MD, based on slowing down the degeneration-regeneration cycles and the metabolic contraction through the inhibition of the transcription factor Nfix.

Nfix, which belongs to the Nuclear factor one (NFI) family, plays an essential role in both prenatal skeletal muscle development and adult regeneration. Indeed, previous studies have reported that Nfix is responsible for the transcriptional switch from embryonic to fetal myogenesis and for the maintenance of the correct timing of skeletal muscle regeneration upon injury (**Figure 1**). More specifically, Nfix deficiency in adult skeletal muscle not only delays muscle regeneration but also promotes a more oxidative fiber metabolism, which has been reported to protect from damage-induced oxidative stress and degeneration.

**Figure 1:**
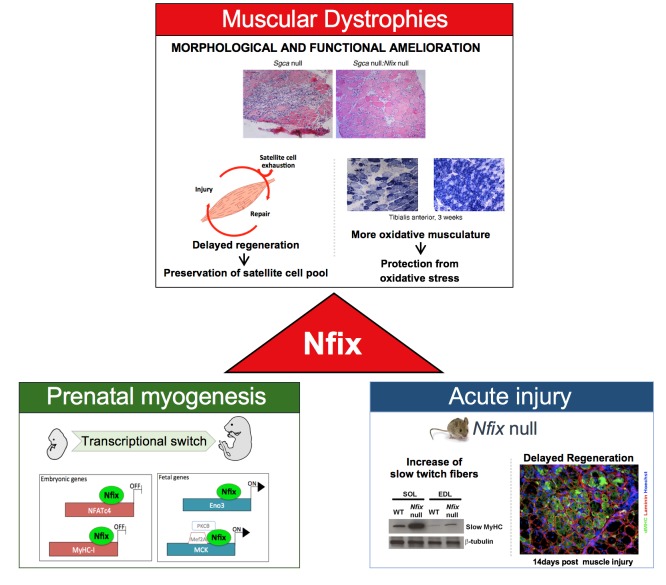
FIGURE 1: Scheme of Nfix functions in muscle development and muscular dystrophy, highlighting how basic biology studies on pre- and post-natal myogenesis built the basis for this translational study opening new therapeutic strategies to treat muscular dystrophies. (adapted from Rossi *et al*., 2016, licensed under the CC BY 4.0 license).

Designed with the aim to identify a common target to treat MDs, our recent work suggests that it is possible to tackle the mechanism of degeneration-regeneration, which is shared by different MDs, through silencing of the transcription factor Nfix.

We explored the role of Nfix in MDs by crossing *Nfix *null mice with different dystrophic mouse models. In many of the experiments, we used *α-sarcoglycan *null mice, a model for human limb-girdle muscular dystrophy 2D, which was chosen for its marked severity and for the presence of a high inflammatory and fibrotic component, similarly to what is observed in DMD patients. However, results were also confirmed in *mdx* mice, the mouse model for Duchenne MD, supporting the broad potential of our approach.

Dystrophic mice lacking Nfix were compared to age-matched dystrophic controls and their histological phenotype was analysed at different time points, up to six months of age. In the absence of Nfix, mice showed an impressive amelioration of the dystrophic signs with respect to controls, such as central nucleation, presence of fibers with varying calibre, inflammatory infiltrates, fibrosis and sarcolemmal integrity. Most importantly, when performance was tested in a run to exhaustion treadmill test, *Nfix* null dystrophic mice showed an increased resistance to fatigue, enabling us to connect the improvement at the histological level with an improvement at the functional level. As hypothesized, this morphological and functional amelioration was associated to delayed muscle regeneration and a general increase in the oxidative fibers. Conversely, overexpression of Nfix in a dystrophic context markedly exacerbated the pathology, further supporting the idea that Nfix can be a valid target for a possible therapeutic strategy.

As proof of principle for the design of new therapeutic interventions, we decided to test our approach by silencing Nfix in adult mice already showing pathological signs, similarly to what would happen in patients. Electroporation of a plasmid silencing Nfix in skeletal muscles of adult dystrophic mice led to a rescue of the histological signs of damage when compared to muscles injected with control plasmids.

This work provided a solid proof of principle to propose an innovative therapeutic approach based on the idea that slowing down the degeneration-regeneration cycles, instead of increasing regeneration, delays the progression of the pathology. Notably, these data also identify Nfix as a key factor in the progression of MD and suggest Nfix as a novel target to treat muscular dystrophy. We hypothesize that blocking Nfix function in a dystrophic context could lead to a more preserved musculature, an increased patients’ life expectancy, and a broader group of patients eligible for clinical trials aiming to correct the genetic defect at the basis of MDs.

We are now developing this ambitious objective by discovering and developing compound(s) able to inhibit Nfix function and/or expression through parallel and highly complementary approaches. These would imply the knowledge of the 3D protein structure of Nfix as also the signalling upstream of Nfix expression. Moreover, a high-throughput screening of small molecule ligands that might interfere with Nfix will contribute to the identification of pharmacological inhibitors of Nfix that would lead to a morphological and functional rescue of the progression of MDs.

